# Ozone Therapy in the Integrated Treatment of Female Dogs with Mammary Cancer: Oxidative Profile and Quality of Life

**DOI:** 10.3390/antiox13060673

**Published:** 2024-05-30

**Authors:** Laís Pereira Silva, Ricardo Wagner Portela, Marília Carneiro Machado, Gisele André Baptista Canuto, João Moreira Costa-Neto, Vitor de Moraes Pina de Carvalho, Hanna Carvalho de Sá, Karine Araújo Damasceno, Vinicius Ricardo Cuña de Souza, Clarisse Simões Coelho, Alessandra Estrela-Lima

**Affiliations:** 1 Research Center on Mammary Oncology (NPqOM), Federal University of Bahia, Salvador 40170-110, Brazil; laisps@ufba.br (L.P.S.); marilia.carneiro@ufrb.edu.br (M.C.M.); jmcn@ufba.br (J.M.C.-N.); vitor.moraes@ufba.br (V.d.M.P.d.C.); karine.damasceno@fiocruz.br (K.A.D.); 2 Laboratory of Immunology and Molecular Biology (LABIMUNO), Institute of Health Sciences, Federal University of Bahia, Salvador 40110-100, Brazil; 3 Center for Agricultural, Environmental and Biological Sciences, Federal University of Reconcavo of Bahia, Cruz das Almas 44380-000, Brazil; 4 Department of Analytical Chemistry, Institute of Chemistry, Federal University of Bahia, Salvador 40170-110, Brazil; gisele.canuto@ufba.br (G.A.B.C.); carvalho.hanna@ufba.br (H.C.d.S.); 5 Department of Anatomy, Pathology and Veterinary Clinics, Federal University of Bahia, Salvador 40170-110, Brazil; 6 Experimental Pathology Laboratory (LAPEX), Gonçalo Moniz Institute, Oswaldo Cruz Foundation (FIOCRUZ), Salvador 40296-710, Brazil; 7 Faculty of Veterinary Medicine, Lusofona University, 1749-024 Lisbon, Portugal; vinicius.souza@ulusofona.pt (V.R.C.d.S.); clarisse.coelho@ulusofona.pt (C.S.C.); 8 Veterinary and Animal Research Centre (CECAV), Faculty of Veterinary Medicine, Lusofona University, 1749-024 Lisbon, Portugal; 9 Mediterranean Institute for Agriculture, Environment and Development, Universidade de Évora, 7004-516 Évora, Portugal

**Keywords:** breast cancer, chemotherapy-induced side effects, integrative medicine, ozone, reactive oxygen species

## Abstract

Considering the high frequency of malignant breast tumors, there is a growing search for new therapeutic strategies that control neoplastic growth and dissemination, combined with fewer adverse reactions. Therefore, this study evaluated the effects of ozone therapy in female dogs with mammary cancer undergoing chemotherapy treatment. Twenty-five canines diagnosed with malignant mammary neoplasia were divided into two groups: one treated with carboplatin alone (*n* = 11) and the other with carboplatin associated with ozone therapy (*n* = 14). Clinical and laboratory evaluations, mastectomy, analysis of the oxidative profile based on total antioxidant capacity (TAC) and serum concentrations of malondialdehyde (MDA), survival rate, and quality of life were performed. Animals in the ozone therapy group had higher concentrations of red blood cells and platelets, significantly improving the survival rate and quality of life. Furthermore, adverse reactions were less intense and frequent in this group, which was associated with an increase in TAC and a reduction in MDA. These results indicate that the combination of carboplatin and ozone therapy represents a promising complementary treatment for female dogs with mammary cancer, as it was associated with fewer adverse reactions and a better oxidative profile.

## 1. Introduction

Breast cancer is one of the most prevalent cancers in women worldwide [1]. Despite substantial advancements in conventional treatments, there is a growing demand for innovative therapeutic strategies that can control neoplastic growth and dissemination along with fewer adverse reactions, especially in patients at advanced stages of the disease [2,3]. In this context, adjuvant therapy using the gas mixture of oxygen–ozone (O_2_–O_3_) emerges as a promising alternative for the treatment of various types of cancer due to its immunomodulatory, anti-inflammatory, and oxidative stress-inducing properties [4,5].

In breast cancer, oxidative stress mechanisms play a fundamental role in activating cellular signaling pathways, proliferation, and apoptosis, influencing tumor progression and aggressiveness [6,7]. In vitro and preclinical studies demonstrate the ozone’s ability to damage tumor cells directly, enhancing the effects of radiotherapy and chemotherapy and reducing adverse reactions to oncological treatments [8,9,10,11]. Additionally, indirect effects have been observed in animal models, such as immunomodulation and tissue oxygenation improvement, as additional benefits during cancer treatment [9,12,13,14].

In women undergoing mastectomy, ozone–oxygen therapy has been shown to accelerate the healing process, enabling radiotherapy application [4]. Preliminary results also suggest that ozone therapy may provide effective therapeutic support in relieving pain and fatigue in patients with various types of cancer during palliative therapies [13,15]. Recent studies investigated the potential of ozone therapy to inhibit the growth of human breast cancer cells, with promising results in inducing apoptosis and inhibiting proliferation [16]. However, the efficacy of ozone therapy as an adjuvant option in oncological treatment requires targeted clinical trials for specific tumors and well-defined circumstances [17].

In veterinary medicine, the use of ozone–oxygen mixture is widespread in various species [18,19], but its application as adjuvant antineoplastic therapy in female dogs, which are considered models for comparative studies on human breast cancer, remains limited. Considering this knowledge gap, the importance and the necessity of new research in this field are evident. In this way, the present study aimed to evaluate the effects of medicinal ozone as an integrative therapy for female dogs with mammary cancer undergoing conventional chemotherapy with carboplatin.

## 2. Materials and Methods

### 2.1. Ethical Aspects

The Ethics Committee for the Use of Experimental Animals of the School of Veterinary Medicine and Zootechny of the Federal University of Bahia approved this research project (protocol n° 18/2021). All the procedures were conducted according to the Brazilian College of Animal Experimentation (COBEA). All animals were domiciliated, and dog guardians were informed about the details of the research project by means of a signed Free and Informed Consent Form.

### 2.2. Clinical Evaluation and Mastectomy

The anamnesis was accomplished by a detailed evaluation of physiological parameters, clinical history, and reproductive records. Preoperative clinical evaluation included a complete blood count; serum urea, creatinine, alkaline phosphatase, alanine aminotransferase, ionized calcium, and glucose concentrations; thoracic radiological examination (anterolateral right, anterolateral left, and ventral-dorsal); and a total abdominal ultrasound to assess for metastasis. Clinical stage classification was performed based on tumor size (T), involvement of regional lymph nodes (N), and presence or absence of distant metastases (M) based on the TNM system [20].

The macroscopic evaluation of inguinal and axillary lymph nodes was performed by palpation. Neoplastic involvement was confirmed by histopathological examination of lymph nodes following mastectomy. All the animals underwent a total unilateral mastectomy to remove the inguinal lymph node. All dogs used compressive bandages and post-surgical clothing until the skin stitches were removed. During this period, physiological and behavioral parameters and possible surgical wound complications were evaluated.

### 2.3. Histological Classification and Grade

Fragments of the affected mammary gland, including skin and subcutaneous tissues, were fixed in phosphate-buffered 10% neutral formalin and processed by the routine paraffin embedding technique. Histological sections (4 μm) were stained by the hematoxylin–eosin (HE) method. In all cases, two veterinarian pathologists analyzed duplicate slides. Tumor samples were categorized according to the histopathological diagnosis following the World Health Organization (WHO) criteria and complemented by the Consensus regarding the diagnosis, prognosis, and treatment of canine and feline mammary tumors, 2019 [20]. Tumors initially classified as solid carcinomas were reclassified following the proposal by Nakagaki et al. [21].

The histological grades of the tumors were described by the Nottingham system as modified by Elston and Ellis [22], which evaluates the percentage of tubule formation, nuclear pleomorphism, and the mitotic index. The female dogs with a histopathological diagnosis of malignancy, histopathological Grades II, III, or regional lymph node metastasis were referred for chemotherapy treatment. Based on histopathological diagnoses, the animals were allocated to groups for treatment in a paired manner, seeking to obtain equivalence of histological types between the groups.

### 2.4. Chemotherapy Treatment

Twenty days post-surgery, carboplatin chemotherapy commenced in six sessions, with intervals of 21−28 days between each session. The dogs received a 5 min infusion of carboplatin via the cephalic vein, with doses ranging from 240 to 300 mg/m^2^ per session, depending on the animal’s size [23]. The primary acute dose-limiting toxicity of carboplatin is myelosuppression, particularly neutropenia, and thrombocytopenia, occurring approximately 14 days post-treatment. However, some dogs, especially those under 10 kg, may reach their nadir at 21 days following carboplatin administration [23].

Fourteen days after each carboplatin session (roughly 48 h before the next session), biological samples were collected for urinalysis, complete blood counts, and serum biochemistry analysis. These laboratory tests confirmed the animals’ suitability for the carboplatin-based chemotherapy procedure. Ondansetron (0.2 mg/kg), ranitidine (2 mg/kg), and promethazine (0.1 mg/kg) were administered subcutaneously. All animals in this study followed this protocol, and these medications were prescribed orally for three consecutive days post-chemotherapy, except for promethazine, which was given as a single dose.

### 2.5. Chemotherapy Treatment and Ozone Therapy

Ozone Life’s specific medical ozone generation equipment was used for ozone therapy. The O&L Portable model was coupled to a medical oxygen cylinder, achieving an ozone concentration with more than 40 values for different oxygen flows (1, ¾ = 0.75, ½ =0.5, ¼ = 0.25, and ⅛1/8 = 0.125) L/min, ensuring precise levels of ozone in the gas mixture. This device is certified by the Brazilian National Agency of Sanitary Surveillance (ANVISA 81509100001).

During the entire cycle of chemotherapy with carboplatin, ozone therapy was performed every seven days in the minor autohemotherapy modality (Ahm—20 μg/ O_2_-O_3_), in the proportion of 1 mL of whole blood (without anticoagulant): 1 mL of the mixture (O_3_–O_2_) with intramuscular (IM) application to the VG 14 acupoint, except on the day of chemotherapy treatment. The VG 14 point, called Governor Vessel 14 (Da-Shui), is in a depression in the dorsal midline, between the seventh cervical vertebra and the first thoracic vertebra, in the cranial direction to the highest point of the inter-scapular. This acupoint (VG 14) is commonly used in hemopuncture, with the objective of modulating the immune system, having indications for its use in cases of immunological disorders, thrombocytopenia, anemia, infectious diseases, and chronic diseases. Indeed, the VG14 point was chosen due to its easy access to the application of ozonated autohemotherapy and its history of stimulating the immune system [24,25].

### 2.6. Chemotherapy Adverse Reactions

The animals were monitored weekly via phone calls during chemotherapy and assessed biweekly through clinical exams focusing on potential adverse drug reactions. To evaluate these reactions, we followed the Veterinary Cooperative Oncology Group—Common Terminology Criteria for Adverse Events (VCOG-CTCAE) guidelines [26]. This consensus outlines a grading scale (from 1 to 5) for the severity of chemotherapy-induced adverse reactions (such as vomiting and diarrhea) in dogs and cats, with the following distinctions: Grade 1 (mild): asymptomatic or mild symptoms, only diagnostic observations, no need for therapeutic intervention; Grade 2 (moderate): moderate limitation of daily activities, requires minimal or non-invasive outpatient intervention; Grade 3 (severe or clinically significant, but not immediately fatal): significant restriction in daily activities, hospitalization indicated; Grade 4 (life-threatening): requires urgent therapeutic intervention; and Grade 5: death related to adverse drug reactions [26]. Dog owners provided information and completed weekly reports indicating potential adverse drug reactions.

### 2.7. Assessment of Hematological and Biochemical Parameters

Blood samples, approximately 5 mL, were obtained by puncture of the jugular vein and previous fasting for 10 h and immediately placed in tubes containing EDTA (ethylenediaminetetraacetic acid) for complete blood counts and in tubes containing coagulant gel for determination of serum biochemical profile. The blood counts were performed using an automated blood cell analyzer (ADVIA 60, Bayer HealthCare, Tarrytown, NY, USA), determining the total counts of red blood cells, leukocytes, hemoglobin, packed cell volume, and platelets. The differential leukocyte counts were performed by optical microscopy evaluation of the blood smears, which were prepared at the time of sample collection, and stained using the Rosenfeld technique.

The gel tubes were centrifuged at 3000 rpm at 4 °C for 10 min to obtain the serum and to perform serum biochemistry tests (liver and kidney function analysis) on a Smart 200 automatic biochemical analyzer. The liver function profile included alkaline phosphatase (FA) and alanine aminotransferase (ALT) serum levels. For renal function evaluation, serum urea and creatinine concentrations were determined. The manufacturer’s recommendations were followed. Hematological and biochemical evaluations were carried out pre-surgery. Pre-chemotherapy assessments consisted of blood count, ALT, and creatinine. Patients whose results were not within the reference values for the species were not subjected to chemotherapy.

### 2.8. Sample Collection for Oxidative Profile Evaluation

To analyze the oxidative status, blood was collected from female dogs undergoing therapeutic protocols at three different times: 48 h before the first chemotherapy session (moment A), 48 h before the fourth chemotherapy session (moment B), and 45 days after completion of the treatment with carboplatin (moment C). From each animal, five milliliters (mL) of blood was collected in tubes containing clot activator and centrifuged at 3000 rpm at 4 °C for 10 min. Then, serum aliquots were placed in microtubes and stored at −80 °C until analysis.

### 2.9. Total Antioxidant Capacity (TAC)

The Trolox Equivalent Antioxidant Capacity assay was used to determine total antioxidant capacity (TAC), following the method described by Erel [27]. Briefly, the 2,2′-Azinobis-(3-ethylbenzothiazoline-6-sulfonic acid) (ABTS) radical was generated by the reaction between the diammonium salt ABTS (7.0 mmol L^−1^) and potassium persulfate (2.45 mmol L^−1^) and was kept in the dark at room temperature for 16 h. The ABTS solution was subsequently diluted with Phosphate-Buffered Saline (PBS) solution until it reached an absorbance of 0.70 nm ± 0.05 nm at 734 nm. The TAC determination was performed on a spectrophotometer (VersaMax™ Microplate Reader, Thermo Fisher Scientific, Waltham, USA) at 734 nm, after 6 min of the reaction of 198 µL ABTS diluted in PBS with 2 µL of standard solutions, samples, or blank. Ethanolic solutions of 6-hydroxy-2,5,7,8-tetramethylchroman-2-carboxylic acid (Trolox), in the range 0.25 to 2.00 mmol L^−1^, were used for calibration in triplicate. PBS was used as a blank sample to correct absorbance. The TAC was calculated by determining the decrease in the absorbance and was expressed as relative to the Trolox standard (in mmol L^−1^).

### 2.10. Determination of Malondialdehyde (MDA) Concentration

MDA serum concentrations were determined following the method described by Tang et al. [28]. MDA was produced from the reaction between 1:100 (*v*/*v*) malondialdehyde bis (dimethyl acetal) and 1% (*v*/*v*) sulfuric acid, kept at room temperature for 2 h. The solution was subsequently diluted ten times with 1% (*v*/*v*) sulfuric acid solution. The calibration curve was prepared from a stock solution of MDA (165 mmol L^−1^) in triplicate and within the concentration range of 0.013−6.63 nmol. The samples were prepared by mixing 20 µL of serum samples with 500 µL of sulfuric acid (42 mmol L^−1^) and adding 125 μL of phosphotungstic acid. The mixture was homogenized by vortexing and maintained at room temperature for 5 min, followed by centrifugation (15,000× *g* for 5 min). Amounts of 102 µL of butylated hydroxytoluene (BHT) (2.5 mmol L^−1^) on ice and 98 µL of deionized water were added to the pellet. Two hundred microliters of samples and standard solutions were mixed with 600 µL of thiobarbituric acid (TBA) (prepared in glacial acetic acid). The mixture was then incubated at 95 °C for 1 h, followed by an ice bath for 10 min. The absorbance was measured at 532 nm on a spectrophotometer (VersaMax™ Microplate Reader, Thermo Fisher Scientific, Waltham, USA). The total concentration of MDA was expressed in nmol mL^−1^.

### 2.11. Assessment of Quality of Life

During the chemotherapy treatment and for 45 days following its conclusion, the quality of life of the female dogs was evaluated using a specific questionnaire completed by their owners. This questionnaire, developed according to the guidelines of Yazbek and Fantoni [29], comprised 12 questions with four possible response options, each ranging from zero to three points, culminating in a maximum score of 36 points. A score of zero indicated the poorest quality of life, while a score of 36 represented the best. The questions focused on behavioral aspects, interaction with the owner, and assessments of pain, appetite, sleep disturbances, vomiting, diarrhea, urinary incontinence, or repletion.

### 2.12. Follow-Up and Survival

Clinical and laboratory tests were conducted every 15 days, while thoracic radiological examinations and total abdominal ultrasounds were performed monthly. One month after completing the conventional chemotherapy cycle with carboplatin (six sessions), the animals underwent clinical evaluations through laboratory tests, which showed that their neutrophil, platelet, and red cell counts were within normal ranges. This follow-up continued until the end of this study or the animal’s death. Specific survival (SS) was defined as the period (in days) from mastectomy to death due to disease progression. In contrast, overall survival time was defined as the period (in days) from the surgical removal of the primary tumor to the date of death (from any cause) or this study’s end date. The medical team evaluated the necessity for humane euthanasia and performed it with the owners’ consent [30]. Animals that died during the follow-up period were necropsied to determine the cause of death and to identify any potential metastases or chemotherapy-induced lesions.

### 2.13. Statistical Analysis

The Student’s *t*-test was applied for independent samples when age and weight means were compared between groups. Analysis of variance for repeated measures (one-way ANOVA) followed by the Tukey test were used to compare different time moments. The Chi-square test and the linear-by-linear association were used for categorical variables to compare proportions between groups. Kaplan–Meier analysis was used to construct the survival curves, and the log-rank test was used to compare the curves from different groups. The Spearman correlation analysis was applied to analyze possible correlations between the evaluated parameters. Binary logistic regression analysis served to compare multiple parameters. Cox regression was used for multivariate survival assessment. The significance level adopted was *p* < 0.05 at a 95% confidence interval, with a two-tailed analysis. Analyses were performed using Prism 8.0 (GraphPad, San Diego, CA, USA) and SPPS 26.0 for Windows (SPSS Inc., Chicago, IL, USA) software.

## 3. Results

### 3.1. Clinical and Pathological Features

From 50 eligible animals with mammary tumors, 25 female dogs diagnosed with malignant mammary neoplasms were included in the current investigation. The age of female dogs in this study ranged from three to 14 years, with a higher frequency of female dogs between 6 and 10 years (14/25—56%) and an average age of 10.2 ± 2.5 years. Weight ranged from 3 to 22 kg with an average of 9.9 ± 4.9, with a predominance of mixed breed animals (SRD) regardless of the group (9/25—36%), followed by Dachshund dogs (4/25—16%) and Poodles, respectively (4/25—16%).

Female dogs with T2 (between 3−5 cm/32% (8/25) and T3 (greater than 5 cm/ 32% (8/25) tumors represented together 64% (16/25) of cases, while animals with metastasis on regional lymph nodes represented 20% (5/25) and with distant metastasis only 8% (2/25). Despite the different types and histological grades of mammary cancer studied, a balance can be seen between the groups evaluated (Table 1). The female dogs that died presented confirmed metastatic foci after necropsy, and the histopathological evaluations of the organs were reclassified to stage V due to a worse prognosis, regardless of the regional metastasis.

For the histopathological grading of the tumors, areas of invasiveness were used, with 16 tumors classified as Grade II and one tumor as Grade I, the latter being a carcinoma in a mixed tumor with behavior considered as atypical and aggressive, since the dog, at the time of diagnosis, already had distant metastasis (Table 2).

### 3.2. Chemotherapy Adverse Reactions

**Figure 1:**
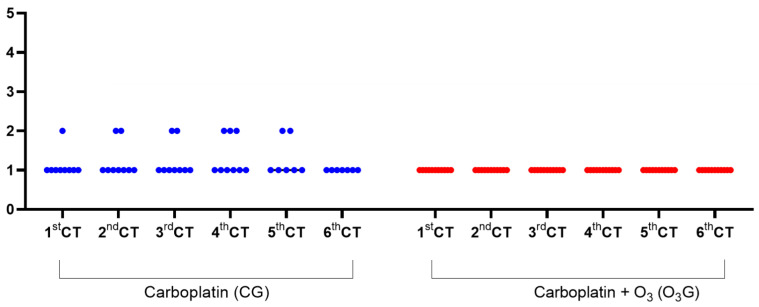
Comparison of degrees of adverse effects in each chemotherapy session according to the type of chemotherapy protocol established in female dogs with malignant mammary neoplasms. Mild, (2) moderate, (3) severe or clinically significant but not immediately fatal, (4) life-threatening, and (5) death related to an adverse drug reaction. The animals were treated with carboplatin alone or with the association of carboplatin and ozone therapy during six chemotherapy sessions. The Tukey test was applied to compare the medians between groups for each CT session and showed no significant difference (*p* < 0.05) between groups in all chemotherapy sessions.

Among the adverse effects/reactions of chemotherapy, regardless of the group, 28% (7/25) of the dogs presented vomiting and 20% (5/25) diarrhea. In the group treated only with carboplatin, Grade 1 adverse reactions were predominant, that is, asymptomatic animals or mild signs, with only diagnostic observations and no indication of therapeutic intervention. However, some animals in the first five CT sessions also presented adverse reactions classified as moderate (Grade 2), with moderate limitation of daily activities and the need for minimal or non-invasive outpatient intervention. In the O_3_G, only reactions classified as mild (Grade 1) were identified (Figure 1). The Tukey test was applied to compare the medians between groups for each CT session. No significant difference was observed between the degrees of adverse reactions between the groups. However, when the Chi-square test was applied, comparing the effects frequencies between the two groups and considering all sessions, a difference was found between the groups (Table 3).

### 3.3. Laboratory Parameters

**Figure 2:**
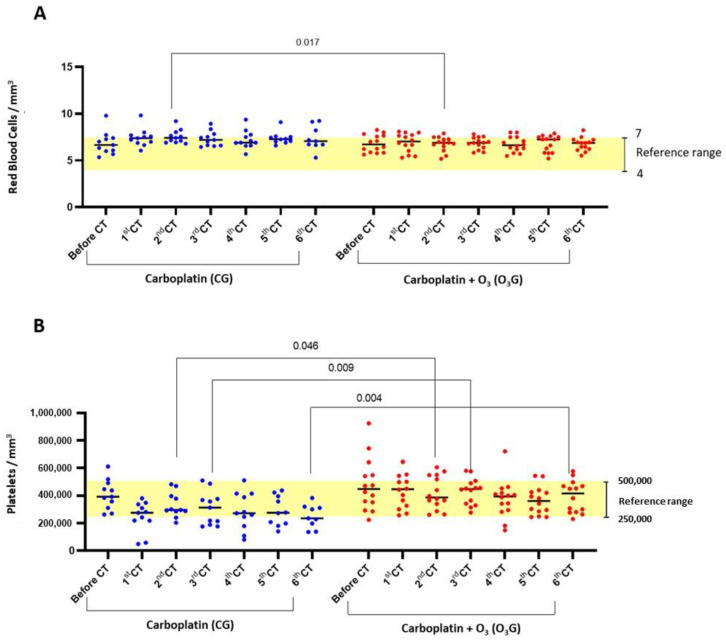
Comparison of red blood cell and platelet counts throughout the chemotherapy treatment of the animals from the control and ozone groups. (**A**) Total red blood cells; (**B**) platelets. The one-way ANOVA statistical test was used, with significance at *p* < 0.05.

Based on hematological parameters, the main effects of chemotherapy were thrombocytopenia and leukopenia due to neutropenia, which were significantly more intense in the group treated with carboplatin alone. The erythrogram of the animals in the O_3_ group revealed a significant increase in red blood cells (*p* = 0.017) after the second CT session, remaining within normal parameters for the species throughout the cycle, and in platelets after the second (*p* = 0.046), third (*p* = 0.009), and sixth (*p* = 0.004) CT sessions when compared to the CG (Figure 2A,B).

**Figure 3:**
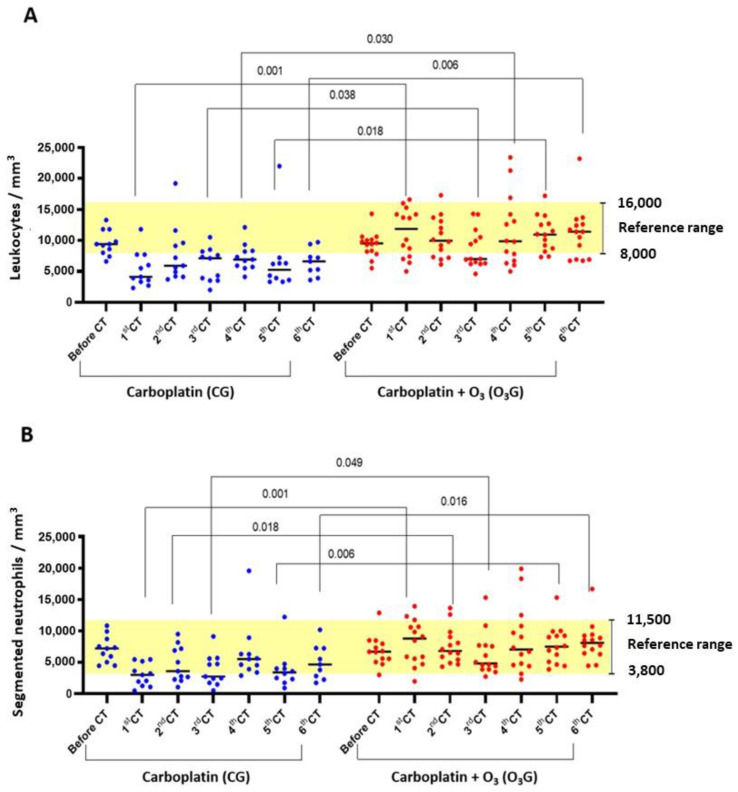
Comparison of total leukocyte and neutrophil counts throughout the chemotherapy treatment. (**A**) Total leukocytes; (**B**) neutrophil granulocytes (segmented neutrophils). The one-way ANOVA statistical test was used, with significance at *p* < 0.05.

Regarding the leukogram, leukopenia due to neutropenia was observed at different times, with a significant difference and lower values found in the carboplatin group (CT). Total leukocytes were reduced after the first (*p* = 0.001), third (*p* = 0.038), fourth (*p* = 0.030), fifth (*p* = 0.018), and sixth CT sessions (*p* = 0.006). Except after the fourth chemotherapy session, there was a significant difference in segmented neutrophil counts throughout the treatment, namely after the first (*p* = 0.001), second (*p* = 0.018), third (*p* = 0.049), fifth (*p* = 0.006), and sixth (*p* = 0.016) CT sessions (Figure 3A,B). Given these results in the group of animals treated only with carboplatin, extending the time between chemotherapy sessions to 28 days was necessary, which was initially established at 21 days. In this study, the biochemical parameters evaluated (creatinine and ALT) in both treated groups were similar.

### 3.4. Quality of Life

After the chemotherapy was carried out on 25 animals, 16 (64%) had their quality of life improved after the start of treatment, five (20%) showed no difference, and two (8%) had the quality of life worsened. In the group undergoing chemotherapy treatment associated with ozone, ten owners (10/14) thought there was an improvement in quality of life (72%), and four owners (28%) were unable to answer the question.

At the beginning of treatment, there was no significant difference between the treatment groups, but after the first chemotherapy session, there was a difference between the groups. In the second session, a difference in quality of life was observed between the groups, and this situation was also seen in the fourth, fifth, and sixth chemotherapy sessions (Table 4). Therefore, comparing the groups separately in terms of the initial and final moments, significant differences in quality of life were observed within the same group in different moments and between groups in the same moment.

**Figure 4:**
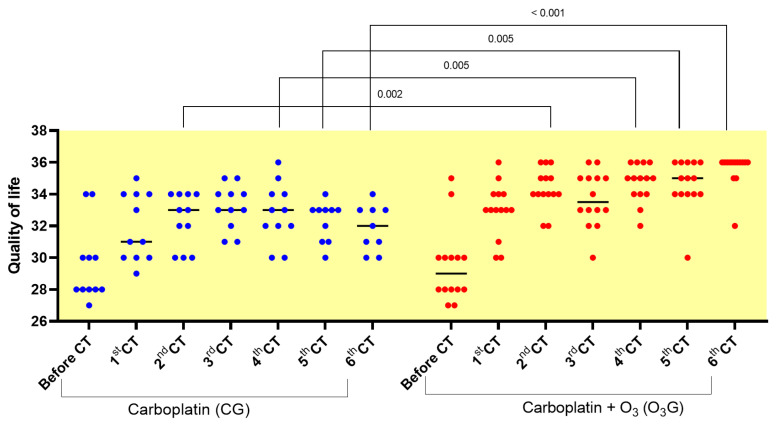
Comparison of quality of life scores between groups in each chemotherapy session. The one-way ANOVA statistical test was used, with significance set at *p* < 0.05.

An increase in the quality of life was observed between the two treatment groups, with greater evidence and significance in the O_3_G when compared to the CG in the second (*p* = 0.002), fourth (*p* = 0.005), fifth (*p* = 0.005), and sixth (*p* < 0.001) CT sessions (Figure 4).

### 3.5. Oxidative Profile—Total Antioxidant Capacity (TAC)

**Figure 5:**
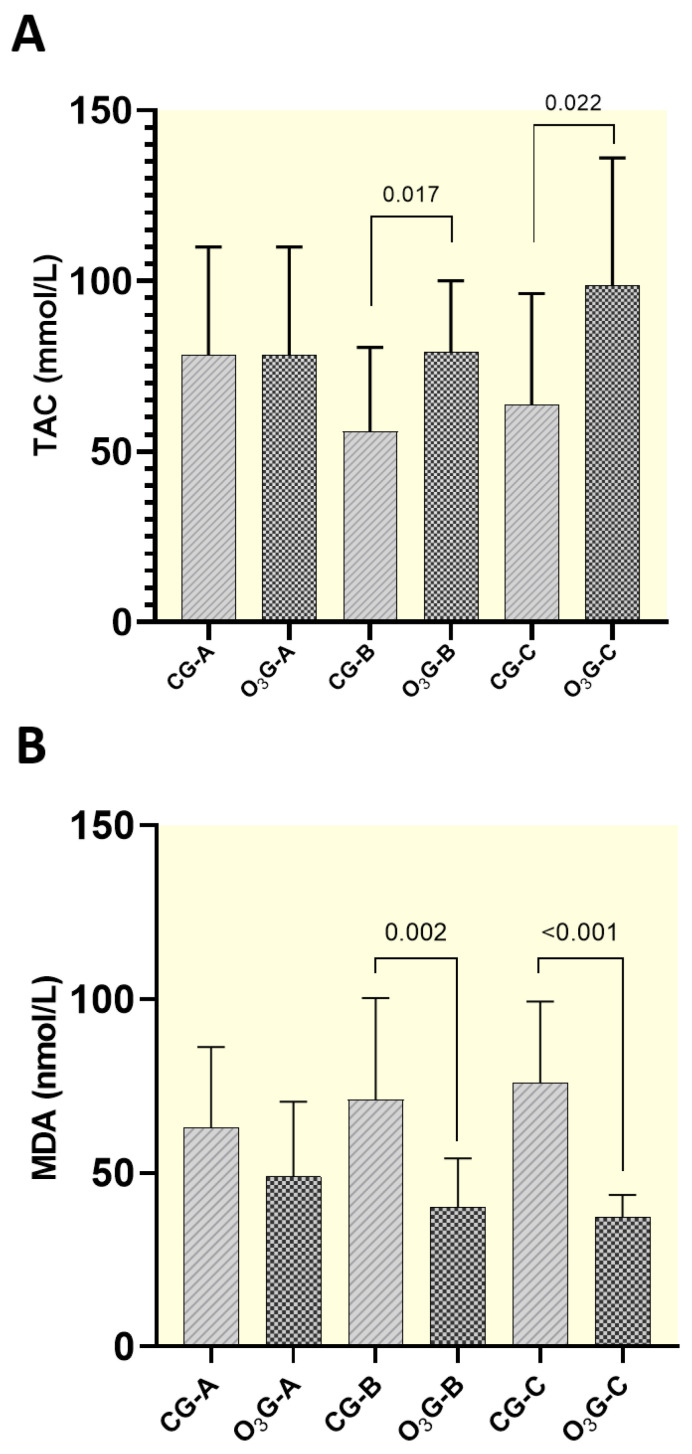
Total antioxidant capacity (TAC) (**A**) and mean serum malondialdehyde (MDA) (**B**) concentrations (nmol/L) of the animals treated with carboplatin alone (CG) or associated with carboplatin and O_3_ (O_3_G) at different times (A, B, and C). Moment A: 48 h before the first chemotherapy session; moment B: 48 h before the fourth chemotherapy session; and moment C: 45 days after completion of the treatment with carboplatin. The one-way ANOVA statistical test was used, with significance at *p* < 0.05.

The mean serum concentrations of total antioxidant capacity (TAC) (mmol/L) between the CG and O_3_G were significantly different (*p* < 0.05). There was no significant difference between the groups in moment A (start of treatment—before the first chemotherapy session). However, at moment B (middle of treatment—moments before the fourth chemotherapy session) and moment C (45 days after the end of therapy), a significant difference was observed (*p* = 0.017 and *p* = 0.022, respectively), with greater total antioxidant capacity in the O_3_G (*p* < 0.05) (Table 5). Additionally, the graph showed an increase in total antioxidant capacity in the group treated with ozone (O_3_G) when compared to the group treated with carboplatin alone (CG), as the treatment progressed (Figure 5A).

### 3.6. Oxidative Profile—Malondialdehyde (MDA)

The mean serum concentrations of malondialdehyde (MDA) (nmol/L) between the CG and O_3_G showed significant differences (*p* < 0.05), except in moment A (start of treatment—before the first chemotherapy session). At moment B (middle of treatment—moments before the fourth chemotherapy session) and moment C (45 days after the end of treatment), there was a significant difference (*p* = 0.002 and *p* < 0.001, respectively) (Table 6). The graph shows a reduction in the concentration of malondialdehyde in the group treated with ozone (O_3_G) when compared to the group treated only with carboplatin (CG) (Figure 5B).

### 3.7. Comparison of Survival Curves

**Figure 6:**
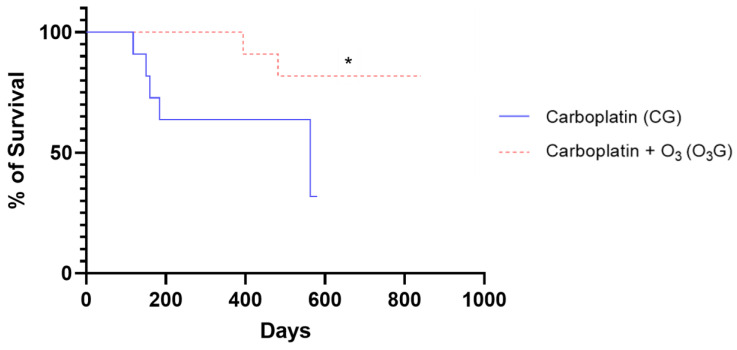
Survival curves of female dogs from the carboplatin group (CG) and carboplatin + O_3_ group (O_3_G) after the chemotherapy sessions. The log-rank (Mantel–Cox) statistical test was used to compare the curves, with significance set at *p* < 0.05, and a significant difference was found (*p* = 0.0224), represented by the symbol “*”.

The maximum follow-up period was approximately 28 months. The minimum survival time was 150 days, attributed to a dog in the carboplatin group with a histological diagnosis of carcinosarcoma that died due to disease progression after the fifth session of chemotherapy. The maximum survival time was 841 days, attributed to a dog from O_3_G with a histological diagnosis of Grade II papillary carcinoma and without lymph node metastasis. This dog was alive and under follow-up at the end of this study. Only the carboplatin group reached the median (563 days), as more than 50% of the population died. Based on the survival of the evaluated groups, significantly higher values can be seen in the O_3_G when compared to the CG (*p* = 0.0224, HR 6.294, and 95% CI 1.252–31.6) (Figure 6).

### 3.8. Univariate and Multivariate Analyses

The analysis of the influence of pathological and clinical parameters associated with survival revealed a significant negative correlation between quality of life and survival in the univariate analysis (*p* = 0.022), indicating that a better quality of life may be associated with a higher chance of survival. However, the multivariate analysis did not maintain this correlation (*p* = 0.170), suggesting that other factors may influence the relationship between quality of life and survival. Ozone therapy (*p* = 0.044) and total antioxidant capacity (TAC) (*p* = 0.039) showed a significant positive correlation with survival only in the multivariate analysis. Meanwhile, malondialdehyde (MDA) showed a significant correlation with survival in both univariate and multivariate analyses (Table 7). This situation suggests that this parameter related to oxidative stress may play an important role in determining survival in female dogs with malignant mammary tumors.

Furthermore, the evaluation of clinical, pathological, and hematologic parameters of ozone therapy demonstrated significant influences on total leukocytes (*p* = 0.017), segmented neutrophils (*p* = 0.046), quality of life (*p* < 0.001), survival (*p* = 0.007), and MDA levels (*p* < 0.001) in both univariate and multivariate analyses. In contrast, total antioxidant capacity (TAC) (*p* = 0.039) exhibited a significant positive correlation with the treatment only in the multivariate analysis (Table 8).

## 4. Discussion

Many desirable effects of chemotherapy arise from increased free radicals and reactive oxygen species (ROS) in the tumor microenvironment. However, they can also mediate chemotherapy-induced toxicity in healthy cells, resulting in important adverse reactions and reduced response to treatment [31]. In this context, this work evaluated the effects of using medicinal ozone as an integrative therapy on the oxidative profile, survival, and quality of life of female dogs with mammary cancer undergoing conventional chemotherapy with carboplatin.

Carboplatin is one of the drugs that are used for the adjuvant treatment of mammary neoplasm in female dogs, resulting in a significant increase in survival rates and quality of life [30,32,33]. It is less nephrotoxic than cisplatin, and vomiting and diarrhea are less frequent and intense during a carboplatin treatment [34]. Machado and collaborators [33], when evaluating the effect of metronomic cyclophosphamide on the tolerability, efficacy, and pharmacokinetics of carboplatin in bitches with mammary carcinoma, reported Grade 1 and 2 adverse reactions in the group of female dogs treated only with carboplatin. Similar results were observed in our study in relation to the carboplatin group; however, in the group of bitches treated with carboplatin associated with ozone therapy, only adverse reactions considered mild (Grade 1) and self-limiting without the need for drug intervention were observed. In addition, a significant reduction in the incidence of nausea and vomiting was detected. It is known that using ozone in adequate doses promotes a reduction in chemoresistance and enhances the action of chemotherapy [10]. Furthermore, ozone can induce adequate doses of antioxidants, reducing or preventing oxidative damage caused by chemotherapy, and providing a clinical benefit for patients undergoing oncological treatment [15,31].

Leukopenia, anemia, and thrombocytopenia of varying intensities are expected adverse reactions in animals undergoing chemotherapy with carboplatin [30,33]. However, interestingly, in the group treated with carboplatin and ozone, a significant increase in the average values of red blood cells, platelets, and leukocytes was observed, including during the nadir period of the drug [23]. In humans, repeated and controlled therapeutic ozone sessions, as major autohemotherapy, induce mild and transient controlled oxidative stress, which results in an increase in the number of red blood cells with improved biochemical and functional characteristics [35,36]. More specifically, in red blood cells stimulated by ozone, there is a transient increase in the production of intracellular ATP with an increase in the rate of glycolysis, leading to the stimulation of the Krebs cycle through the production of 3-phosphoglycerate by 2,3 diphosphoglycerate (2, 3 DPG) and glucose 6 phosphate dehydrogenase (G6PDH), with changes in the hemoglobin dissociation curve, increased oxygen supply to tissues, in addition to higher concentrations of antioxidant enzymes [36,37].

Also, ozone can influence the rheological properties of blood; that is, it can improve the flexibility and elasticity of erythrocyte membranes and reduce blood viscosity [37,38]. There is also induction of nitric oxide production by vascular endothelial cells and, thus, promotion of vasodilation at the microcirculation level. In this way, activated erythrocytes enter the circulation gradually and continuously, replacing old erythrocytes adapted to chronic oxidative stress in a process known as oxidative preconditioning, as they regulate the antioxidant system [9,39]. Although Bocci et al. [9] suggest that 15 sessions of autohemotherapy are necessary to stimulate the production of new erythrocytes with biochemical improvements induced by ozone, in the present study, a significant increase in erythrocyte values was observed in the second session and, consequently, in tissue oxygenation in animals in the O_3_G.

The assessment of tumor hypoxia was not performed in this study; however, it is noteworthy that increased concentrations of red blood cells in O_3_G can result in better blood flow, greater transfer of oxygen to hypoxemic tissues, and a consequent better response to treatment. It is known that tumor hypoxia is an adverse factor for chemotherapy and radiotherapy, as it induces a physiological selection of tumor cells with reduced apoptotic potential, resulting in resistance to treatment and greater potential for tumor aggressiveness [40,41].

Another interesting result refers to the significant increase in the number of platelets observed after the first chemotherapy session in animals from the carboplatin and ozone group. The effects caused by ozone therapy on platelet induction are still controversial, and data in the literature regarding this process are scarce [35,42]. Nevertheless, Valacchi and Bocci [43] reported an increase in the number of platelets, reduced platelet aggregation, significantly greater amounts of platelet-derived growth factor (PDGF), and transforming growth factor-beta 1 (TGF-beta1) and interleukin-8 (IL-8), which are dose-dependent and released after ozonation, at a concentration of 20 µg O_3_/mL to 80 µg O_3_/mL, by heparinized platelets in human blood samples. The ozone concentration in the present study is within the therapeutic window proposed by the aforementioned authors and justifies the increase in the number of platelets and lower platelet aggregation in the group that received weekly minor ozonated autohemotherapy.

Regarding the leukocyte counts, leukopenia was found in the CG animals, as expected, while a significant increase in total leukocytes and segmented neutrophils was identified in the animals that underwent weekly minor ozonated autohemotherapy. Ozone, through the transient action of H_2_O_2_, has a long-lasting and delayed action on polyunsaturated fatty acids (PUFAs) and other antioxidants, acting as a modulator of the immune system by inducing the production of cytokines in neutrophils, lymphocytes, and monocytes, which can cause leukocytosis and activation of leukocytes [9,44,45]. However, it is worth highlighting that, in the present study, the increase in total leukocyte and segmented neutrophil counts did not characterize leukocytosis, given that the values observed were within the reference range for the species, even during the nadir of the drug, when evident leukopenia was expected.

The therapeutic toxicity biochemical markers (ALT and creatinine) evaluated throughout the treatment remained within physiological parameters for the species for most of the treatment, regardless of the group. These results were expected and suggest that carboplatin chemotherapy did not affect the morphological integrity of hepatocytes and renal function. In fact, according to the literature, despite the renal excretion of the chemotherapy drug, carboplatin is a drug considered safe and can be used in nephropathic patients with discretion [46,47].

In the liver function assessment, the maintenance of ALT values is also justified, as carboplatin does not cause liver toxicity [48]. Aslaner and collaborators [49] evaluated the protective effect of ozone therapy on hepatotoxicity induced by methotrexate, a chemotherapy drug with the potential to cause hepatic steatosis, cholestasis, fibrosis, and cirrhosis, and concluded that the association with ozone resulted in a decrease in ALT levels. Absence of changes in serum biochemistry (creatinine and ALT) and hematological changes restricted to the nadir period of the drug in female dogs with breast cancer treated with carboplatin were also reported by Machado et al. [30], Machado et al. [33], and Lavalle et al. [32].

Oxidative stress was evaluated through assays on total antioxidant capacity (TAC) and lipid peroxidation product, measuring the malondialdehyde (MDA) serum levels. The analysis of TAC and MDA revealed linear, inverse, and significantly different profiles between the CG and O_3_G at moments B (48 h before the fourth chemotherapy session) and C (45 days after completion of the treatment with carboplatin). In the group treated only with carboplatin, TAC values were decreasing, and those of MDA increased during chemotherapy treatment; the opposite was observed in the O_3_G as treatment progressed, that is, TAC with an increasing linear profile and decreasing MDA. The increase in MDA in cancer patients, despite being controversial, has been associated with excessive production of reactive oxygen species (ROS) by neoplastic cells, antioxidant deficiency, induction of oxidative stress followed by molecular damage, and lipid peroxidation in different types of cancer, including human and canine breast cancer patients [10,11,50,51,52]. However, the increase in ROS and consequent genetic instability and increase in MDA can also be induced by surgery and mainly by chemotherapy. Some chemotherapy drugs, such as platinum derivatives, including carboplatin, induce apoptosis of neoplastic cells through an increase in ROS, which is one of their main mechanisms of action [13], justifying the increasing and significant MDA values observed in the group treated with carboplatin alone.

Regarding the high TAC and reduced MDA levels in the O_3_G, the results demonstrated a reduction in oxidative stress status with ozone therapy. It is known that ozone acts as a modulator or pro-drug and enhances subsequent adaptive responses by inducing secondary messengers. Hydrogen peroxide, one of these secondary messengers, promotes the nuclear factor 2-related factor erythroid 2 (Nrf2) pathway and the synthesis of proteins that favor cell survival [53,54,55]. Low doses of O_3_ generate mild oxidative stress, which activates the Keap1-dependent pathway mediated by Nrf2, which, in turn, stimulates gene expression of antioxidant response elements (AREs) [55]. Under basal conditions, Nrf2 is close to the Keap 1 protein; when there are stressful stimuli, Nrf2 moves from Keap1 and enters the cell nucleus, inducing the transcription of genes triggered by antioxidant response elements [56]. Ozonation prevents the degradation of Nrf2 and promotes its translocation to the nucleus, activating antioxidant response elements [55]. Experimental results demonstrated that ex vivo or in vivo ozone can activate Nrf2 [53,55]. This mechanism may explain the genomic target of ozone, which induces a response based on protein synthesis, such as the antioxidant enzymes HO-1, SOD, and CAT, providing more effective cellular protection against the harmful effects of free radicals, including lipid peroxidation/elevation of MDA resulting from chemotherapy treatment [53]. The results agree with other studies described in the literature and may suggest that a controlled application of O_3_ during chemotherapy can protect against prolonged and important oxidative stress [31].

An increase in quality of life was observed in the O_3_G, with an average quality of life score close to 36, which is considered the best possible index. The results obtained from the analysis of the questionnaires were confirmed by the satisfactory clinical response and reports from the tutors regarding the better general condition of the animals after ozone therapy. In a previous study carried out by our group, female dogs submitted only to chemotherapy with carboplatin presented quality of life scores ranging from 20 to 23, while those treated with chemotherapy and low-dose naltrexone (LDN) ranged from 31 to 36 [30]. Therefore, the results of this study support the idea that quality of life is directly related to the beneficial effects of ozone. Similarly, human clinical studies have demonstrated improved quality of life, reduced levels of reactive oxygen species (ROS), and increased antioxidant potential in patients who received chemotherapy combined with ozone therapy [15,31].

Of the seven dogs that died because of the neoplastic disease, five belonged to the group treated with carboplatin alone, and two belonged to the group treated with carboplatin associated with ozone therapy. The higher survival rate observed in the O_3_G is a reflection, among other factors, of the better quality of life. Recently, the effect of ozone treatment on health-related quality of life and toxicity induced by radiotherapy and chemotherapy in symptomatic cancer survivors was also evaluated [57]. The results demonstrated that ozone treatment has the potential to positively impact not only the treatment, as observed in our study, but also the care of cancer survivors undergoing radiotherapy and chemotherapy by significantly improving quality of life, reducing toxicity, and offering a promising therapeutic option for managing chronic side effects.

We acknowledge the inherent limitations of our study, particularly regarding the small sample size and the heterogeneity of the tumors in terms of diagnosis and histopathological grading. These limitations precluded a more robust comparison with previously reported results observed in women, especially concerning a specific type of tumor. However, considering that the analyzed tumors were spontaneous and there was a balance between the evaluated groups, this same heterogeneity may have contributed to demonstrating the potential of ozone therapy as a complementary therapy in clinical practice. This approach is pertinent, given that the diversity of malignant mammary tumor types is a reality in medical practice.

## 5. Conclusions

The promising results of this study, although preliminary, encourage further exploration of ozone therapy as a complementary therapeutic alternative in the treatment of breast cancer. Ozone treatment has demonstrated beneficial or potentially beneficial effects by reducing adverse reactions to chemotherapy, promoting positive modulation of the oxidative profile, and improving quality of life, leading to increased survival rates in dogs undergoing treatment for mammary cancer. However, clinical trials involving more animals are necessary to validate the results observed herein and confirm the efficacy and safety of ozone therapy within the context of veterinary, comparative, and translational oncology.

## Figures and Tables

**Table 1:** Clinical and pathological characteristics of the female dogs with mammary neoplasms included in this study.

	Carboplatin (CG)	Carboplatin + O_3_ (O_3_G)	Total	
	Mean (±SD)	Mean (±SD)	Mean (±SD)	*p*-Value
**Weight** **(Kg)**	10.8 (±1.9)	9.2 (±3.7)	9.9 (±4.9)	0.428
Minimum	3.9	3	3	
Maximum	22	15.3	22	
**Variable**	**n (%)**	**n (%)**	**n (%)**	
**Size**				0.468
Mini	2 (18.2)	2 (14.3)	4 (16.0)	
Small	4 (36.4)	9 (64.3)	13 (52.0)	
Average	5 (45.5)	3 (21.4)	8 (32.0)	
**Breed**				0.231
Mixed breed	5 (45.5)	4 (28.6)	9 (36.0)	
Beagle	1 (9.1)	1 (7.1)	2 (8.0)	
Poodle	2 (18.2)	2 (14.3)	4 (16.0)	
Dachshund	1 (9.1)	3 (21.4)	4 (16.0)	
Pinscher	1 (9.1)	1 (7.1)	2 (8.0)	
Yorkshire Terrier	1 (9.1)	1 (7.1)	2 (8.0)	
Maltese	0	1 (7.1)	1 (4.0)	
Shih Tzu	0	1 (7.1)	1 (4.0)	
**Reproductive Status**				0.198
Neutered	2 (18.2)	6 (42.9)	8 (32.0)	
Not neutered	9 (81.8)	8 (57.1)	17 (68.0)	
**Affected Mammary Chain**				0.695
Unilateral	6 (54.5)	6 (42.9)	12 (48.0)	
Bilateral	5 (45.5)	8 (57.1)	13 (52.0)	
**Involvement**				0.21
Single	5 (45.5)	3 (21.4)	8 (32.0)	
Multiple	6 (54.5)	11 (78.6)	17 (68.0)	
**TNM Stage**				0.802
I	0	2 (14.3)	2 (8.0)	
II	5 (45.5)	3 (21.4)	8 (32.0)	
III	3 (27.3)	5 (35.7)	8 (32.0)	
IV	3 (27.3)	2 (14.3)	5 (20.0)	
V	0	2 (14.3)	2 (8.0)	
**Lymph Node**				0.739
Metastasis	3 (27.3)	3 (21.4)	6 (24.0)	
No metastasis	8 (72.7)	11 (78.6)	19 (76.0)	
**Histological Grade**				0.388
Grade I	0	1 (10.0)	1 (5.9)	
Grade II	7 (100)	9 (90.0)	16 (94.1)	

The animals were divided into two treatment groups: one treated only with carboplatin and the second one treated with carboplatin and ozone. Statistical tests: Student’s *t*-test, Chi-square, and linear-by-linear association. Significance set at *p* < 0.05.

**Table 2:** Histopathological diagnosis and grading of mammary tumors in dogs were included in this study.

	Carboplatin (CG)	Carboplatin + O_3_ (O_3_G)	Total	
	n (%)	n (%)	n (%)	
**Histological Types**				0.146
Malignant adenomyoepithelioma	3 (27.3)	1 (7.1)	4 (16.0)	
Carcinoma in a mixed tumor (Grade II)	4 (36.4)	5 (35.7)	9 (36.0)	
Papillary carcinoma (Grade II)	1 (9.1)	2 (14.2)	3 (12.0)	
Solid papillary carcinoma (Grade II)	1 (9.1)	1 (7.1)	2 (8.0)	
Carcinosarcoma	1 (9.1)	2 (14.3)	3 (12.0)	
Malignant myoepithelioma	0	1 (7.1)	1 (4.0)	
Carcinoma in a mixed tumor (Grade I)	0	1 (7.1)	1 (4.0)	
Carcinoma with solid pattern (Grade II)	1 (9.1)	1 (7.1)	2 (8.0)	
**Histological Grade**				0.388
Grade I	0	1 (10.0)	1 (5.9)	
Grade II	7 (100)	9 (90.0)	16 (94.1)	

The animals were divided into two treatment groups: one treated only with carboplatin and the second one treated with carboplatin and ozone. Statistical tests: Chi-square and linear-by-linear association. Significance at *p* < 0.05.

**Table 3:** Comparison of adverse reaction degrees between experimental groups.

	Carboplatin (CG)	Carboplatin + O_3_ (O_3_G)	*p*-Value
Adverse reaction	n (%)	n (%)	<0.001
Mild	47 (73.4)	84 (100)	
Moderate	17 (26.6)	0	

The Chi-square statistical test was used to compare the results between the groups, with significance at *p* < 0.05.

**Table 4:** Comparison of quality of life between treated groups and in each chemotherapy (CT) session.

	Start	1st CT	2nd CT	3rd CT	4th CT	5th CT	6th CT
**Quality of life**							
**CG**	29.5 (±2.4) a	31.9 (±2.1) b	32.4 (±1.7) Ab	33.2 (±1.4) b	32.8 (±1.9) Ab	32.3 (±1.2) Ab	31.9 (±1.4) Ab
**O3G**	29.5 (±2.4) a	33.0 (±1.7) b	34.3 (±1.3) Bb	33.7 (±1.7) b	34.7 (±1.2) Bb	34.6 (±1.6) Bb	35.6 (±1.1) Bb

The results are expressed as means ± standard deviations, and different capital letters demonstrate statistical differences between groups (comparison of results in different lines). In addition, different lowercase letters demonstrate statistical differences between moments (comparison of results in different columns). The Chi-square statistical test was used, with significance at *p* < 0.05.

**Table 5:** Total antioxidant capacity (TAC) (mmol/L) serum concentration means and standard deviations in the CG and O_3_G at different moments (A, B, and C).

TAC (mmol/L)			
	Carboplatin (CG)	Carboplatin + O_3_ (O_3_G)	*p*-Value
**Moments**			
**A**	78.2 (±31.7)	69.6 (±33.0)	0.517
**B**	55.8 (±24.7)	79.2 (±20.7)	0.017 *
**C**	63.5 (±32.7)	98.6 (±37.4)	0.022 *

The symbol “*” expresses statistically significant differences. The one-way ANOVA statistical test was used, followed by the Student’s *t*-test, with significance set at *p* < 0.05. Moment A: 48 h before the first chemotherapy session; moment B: 48 h before the fourth chemotherapy session; and moment C: 45 days after completion of the treatment with carboplatin.

**Table 6:** Estimated means and standard deviations of serum concentrations of MDA (nmol/mL) in the CG and O_3_G in the different moments (A, B, and C).

MDA (mmol/L)			
	Carboplatin (CG)	Carboplatin + O_3_ (O_3_G)	*p*-Value
**Moments**			
**A**	62.8 (±23.4)	48.9 (21.6)	0.136
**B**	71.1 (±29.1)	40.2 (±13.9)	0.002 *
**C**	75.9 (±23.4)	37.3 (±6.4)	<0.001 *

Moment A: 48 h before the first chemotherapy session; moment B: 48 h before the fourth chemotherapy session; and moment C: 45 days after completion of the treatment with carboplatin. The symbol * expresses statistically significant differences. The one-way ANOVA statistical test was used, followed by the Student’s *t*-test, with significance at *p* < 0.05.

**Table 7:** Univariate and multivariate analyses of clinical, pathological, and hematological parameters in female dogs with malignant mammary tumors considering the association with survival.

Parameters	Univariate ^a^		Multivariate ^b^	
	Spearman’s Rho (CI 95%)	*p*-Value	Odds Ratio (CI 95%)	*p*-Value
Ozone therapy	0.268 (−0.081–0.582)	0.196	0.177 (0.033–0.955)	0.044 *
Alanine aminotransferase	−0.173 (−0.529–0.188)	0.506	0.926 (0.834–1.029)	0.152
Creatinine	0.224 (−0.276–0.591)	0.387	2.445 (0.410–14.574)	0.326
Red cells	0.087 (−0.479–0.428)	0.992	0.599 (0.190–1.888)	0.382
Platelets	−0.410 (−0.744–0.155)	0.102	1.006 (0.989–1.011)	0.132
Total leukocytes	−0.284 (−0.652–0.156)	0.270	1.009 (0.995–1.014)	0.179
Segmented neutrophils	−0.409 (−0.716–0.257)	0.103	1.002 (0.968–1.023)	0.081
Quality of life	−0.551 (−0.825–−0.195)	0.022 *	0.732 (0.469–1.143)	0.170
Adverse effects	0.310 (−0.169–0.669)	0.226	3.344 (0.342–32.736)	0.300
Clinical staging	0.278 (−0.038–0.628)	0.280	1.200 (0.637–2.260)	0.572
Tumor grade	0.116 (0.063–0.281)	0.658	2.303 (0.087–12.145)	0.43
TAC	−0.472 (−0.743–−0.158)	0.055	0.971 (0.944–0.998)	0.039 *
MDA	0.567 (0.262–0.796)	0.018*	1.038 (1.004–1.073)	0.026 *

Significant statistical associations are expressed by the symbol * when using the Spearman correlation test (^a^) or the Cox regression test (^b^), with significance at *p* < 0.05.

**Table 8:** Univariate and multivariate analyses of clinical, pathological, and hematologic parameters associated with carboplatin + ozone treatment in female dogs with malignant mammary tumors.

Parameters	Univariate ^a^		Multivariate ^b^	
	Spearman’s Rho (CI 95%)	*p*-Value	Odds Ratio (CI 95%)	*p*-Value
Alanine aminotransferase	−0.383 (−0.761–0.103)	0.143	0.955 (0.907–1.005)	0.078
Creatinine	−0.111 (−0.629–0.449)	0.681	0.246 (0.008–7.491)	0.421
Red cells	−0.342 (−0.754–0.193)	0.195	0.413 (0.141–1.209)	0.106
Platelets	0.410 (−0.104–0.790)	0.114	1.004 (0.947–1.627)	0.115
Total leukocytes	0.588 (0.192–0.855)	0.017 *	1.208 (1.004–1.470)	0.020 *
Segmented neutrophils	0.506 (0.032–0.820)	0.046 *	1.015 (0.992–1.133)	0.037 *
Quality of life	0.923 (0.823–0.988)	<0.001 *	4.594 (1.445–14.607)	0.010 *
Adverse effects	−0.293 (−0.620–−0.174)	0.271	0.290 (0.021–4.026)	0.357
Clinical staging	0.043 (−0.486–0.529)	0.875	1.260 (0.555–2.858)	0.580
Tumor grade	−0.209 (−0.436–−0.139)	0.420	1.778 (0.257–15.273)	0.388
Survival (days) Condition (live)	0.525 (0.191–0.784) −0.345 (−0.678–0.050)	0.007 * 0.092	1.005 (1.001–1.010) 0.200 (0.030–1.351)	0.027 * 0.099
TAC	0.451 (−0.010–0.216)	0.080	1.022 (1.001–1.038)	0.035 *
MDA	−0.861 (0.047–−0.877)	<0.001 *	0.789 (0.627–0.993)	0.043 *

Significant statistical associations are expressed by the symbol * when using the Spearman correlation test (^a^) or the Cox regression test (^b^), with significance at *p* < 0.05.

## Data Availability

The raw data supporting this article’s conclusions are available upon request.
